# Adult cor triatriatum sinistrum: a rare cause of ischaemic stroke

**DOI:** 10.1007/s12471-016-0938-z

**Published:** 2016-12-13

**Authors:** L. Baris, A. J. J. C. Bogers, E. J. van den Bos, M. J. M. Kofflard

**Affiliations:** 1 Department of Cardiology, Albert Schweitzer Hospital, Dordrecht, The Netherlands; 2 Department of Cardio-thoracic Surgery, Erasmus MC, Rotterdam, The Netherlands

A 55-year-old male was referred to the cardiologist because of the occurrence of two ischaemic strokes within five months’ time in the absence of documented atrial fibrillation. At echocardiography, a membrane was visualised in the left atrium (cor triatriatum) (Fig. 1a). Since no other abnormalities were detected, the cor triatriatum was held responsible for the cardioembolic stroke. Surgical resection of the fenestrated membrane was carried out successfully (Fig. 1b).

**Fig. 1:**
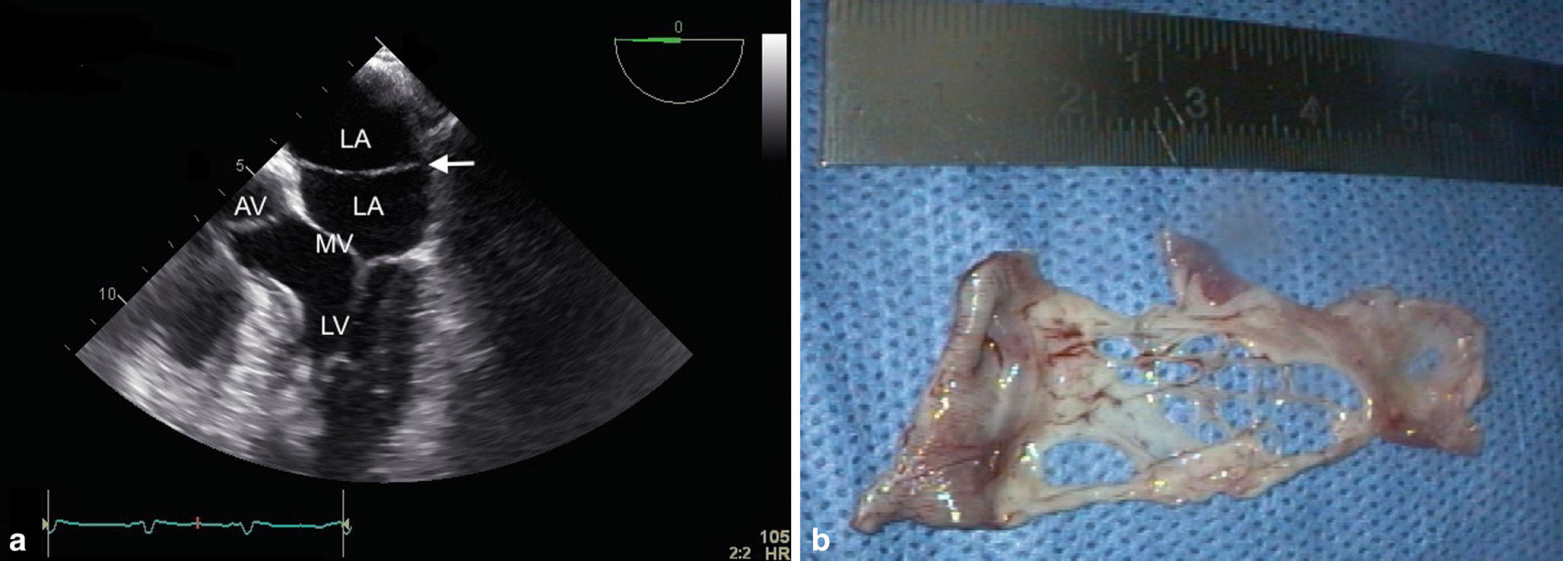
**a** Transoesophageal echocardiographic image of the left atrium (*LA*), the left ventricle (*LV*), aortic valve (*AV*) and the mitral valve (*MV*). The arrow points to the fibromuscular membrane. **b** Postoperative image of the resected fibromuscular membrane with multiple fenestrations

Cor triatriatum is a rare congenital cardiac malformation often recognised during childhood [1] and is usually accompanied by other congenital abnormalities [2]. Symptoms result from the obstructive property mimicking mitral stenosis [3]. In a minority of cases, it is found during routine evaluation in asymptomatic adults. Cor triatriatum as a source of cardioembolic stroke is rare and in most cases atrial fibrillation is an associated finding [2]. Anticoagulant medication or surgery are proposed therapies; however, there is no consensus with respect to the best strategy [4].

## Caption Electronic Supplementary Material

Video 1: In this video one can appreciate the fibromuscular membrane dividing the left atrium in a proximal and distal part

Video 2: In this video with colour Doppler, the fenestrations in the membrane are clearly visible

## References

[1] Niwayama G (1960). Cor triatriatum. Am Heart J.

[2] Eichholz JL, Hodroge SS, Crook JJ, Mack JW, Wortham DC (2013). Cor triatriatum sinister in a 43-year-old man with syncope. Tex Heart Inst J.

[3] Leung KF, Lau AT (2015). Cor triatriatum: a rare cause of embolisation. Hong Kong Med J.

[4] Saxena P, Burkhart HM, Schaff HV, Daly R, Joyce LD, Dearani JA (2014). Surgical repair of cor triatriatum sinister: the Mayo Clinic 50-year experience. Ann Thorac Surg.

